# The Impact of Liposomal Irinotecan on the Treatment of Advanced Pancreatic Adenocarcinoma: Real-World Experience in a Taiwanese Cohort

**DOI:** 10.1038/s41598-020-64421-6

**Published:** 2020-05-04

**Authors:** Yung-Yeh Su, Nai-Jung Chiang, Hui-Jen Tsai, Chia-Jui Yen, Yan‐Shen Shan, Li‐Tzong Chen

**Affiliations:** 1 National Institute of Cancer Research, National Health Research Institutes, Tainan, Taiwan; 2 Division of Hematology and Oncology, Department of Internal Medicine, National Cheng Kung University Hospital, College of Medicine, National Cheng Kung University, Tainan, Taiwan; 3 Institute of Clinical Medicine, College of Medicine, National Cheng Kung University, Tainan, Taiwan; 4 College of Medicine, National Cheng Kung University, Tainan, Taiwan; 5 Department of Internal Medicine, Kaohsiung Medical University Hospital and Kaohsiung Medical University, Kaohsiung, Taiwan

**Keywords:** Gastroenterology, Oncology

## Abstract

Liposomal irinotecan plus 5-fluorouracil/leucovorin (nal-IRI + 5-FU/LV) has shown to provide survival benefits for patients with gemcitabine-refractory metastatic pancreatic ductal adenocarcinoma (PDAC) in NAPOLI-1 trial, in which Asian patients experienced more hematological toxicity and subsequent dose modification. A retrospective chart review to investigate the administration pattern, therapeutic efficacy and safety profile of nal-IRI + 5-FU/LV in 44 consecutive patients with gemcitabine-refractory advanced PDAC treated between December 2016 and December 2018 in National Cheng Kung University Hospital, Taiwan. Most of them had metastatic diseases (88.6%), one-line of prior treatment (72.7%), ECOG PS 0-1 (72.7%) and starting dose of nal-IRI at 60 mg/m^2^ (≈52 mg/m^2^ irinotecan free-base) in 65.9%. The overall response rate was 9.1%. The median OS was 6.6 months for the entire cohort, and 7.8 and 2.7 months for patients of ECOG PS 0-1 and>2, respectively. The median OS of ECOG PS 0-1 patients with nal-IRI starting doses at 80 mg/m^2^ (≈70 mg/m^2^ irinotecan free-base, n = 13) and 60 mg/m^2^ (n = 19) were 7.5 and 8.4 months, respectively. Thirty-four percent of patients experienced manageable grade 3-4 hematological toxicity. Our results confirm the clinical benefit of nal-IRI + 5-FU/LV for patients of gemcitabine-refractory advanced PDAC with good performance status in a real-world setting.

## Introduction

Pancreatic cancer was the seventh leading cause of global cancer mortality in 2018, and predicted to become the second by 2030 in the U.S^1,2^. Despite the recent advance in understanding the biology and multidiscipline management of pancreatic cancer, the 5-year survival rate of all-stage newly diagnosed pancreatic cancer was only 8%^3^.

Majority of patients with pancreatic ductal adenocarcinoma (PDAC), the most common histological type of pancreatic cancer, presented with either unresectable locally advanced or metastatic diseases upon diagnosis. Systemic chemotherapy with gemcitabine has been the standard of care for advanced PDAC since 1997. The efficacy of gemcitabine was not satisfactory, with a median OS of merely 5.7 months in the pivotal trial^4^. However, the progress in systemic chemotherapy for advanced PDAC has been limited until the emergence of FOLFIRINOX (5-FU/leucovorin + irinotecan + oxaliplatin) and the gemcitabine plus nab-paclitaxel regimens in early 2010s. Both regimens provided significant survival benefits over gemcitabine monotherapy, with a median OS of 11.1 *versus* 6.8 months (hazard ratio [HR] = 0.57, p < 0.001) and 8.5 *versus* 6.7 (95% CI: 6.0–7.2) months, HR = 0.72 (P < 0.001), respectively^5,6^, and have currently been recognized as global standard first-line treatments for patients of advanced PDAC with good general condition^7–11^.

Oral S-1, a third generation oral fluoropyrimidine, is another useful and approved agent for the treatment of pancreatic cancer in Asia. S-1 monotherapy has been shown to achieve significantly better response rate and non-inferior overall survival against gemcitabine alone for advanced PDAC patients in the randomized phase III GEST trial^12^. For its favorable safety profile, S-1 either alone or combining with gemcitabine are acceptable regimens for less fit, advanced PDAC patients in Japan^10,13^ and for all comers in clinical practice before the reimbursement of FOLFIRINOX and the nab-paclitaxel in Taiwan. While biweekly triplet chemotherapy consisting of gemcitabine, oxaliplatin plus either infusion 5-FU/LV (the GOFL regimen) or oral S-1/LV (the SLOG regimen) that have been developed through a series of multicenter trials under the platform of Taiwan Cooperative Oncology Group (TCOG) are the favorable regimens in our institutional clinical practice^14,15^. In a single arm phase II trial, SLOG had 40.7% of overall response rate, and 7.6 and 11.4 months of median progression-free survival and overall survival, respectively, that are comparable with those achievable with FOLFIRINOX in ACCORD 11/0402 study^15^.

There was no consensus regarding second-line treatment for pancreatic cancer until 2015, in which global NAPOLI-1 trial showed liposomal irinotecan plus 5-FU/leucovorin (nal-IRI + 5-FU/LV) not only significantly extended survival but also preserved quality of life in patients with gemcitabine-refractory metastatic PDAC as compared to 5-FU/LV^16,17^. Nal-IRI is a new formulation of irinotecan encapsulated in pegylated liposomes which prevents the premature metabolism of irinotecan in the liver and can passively diffuse through leaky tumor vasculature, resulting in favorable circulation and intra-tumor pharmacokinetics of SN-38, the active metabolite of irinotecan, as compared to conventional irinotecan^18,19^.

Aside from the pivotal NAPOLI-1 trial, real-world data reporting the efficacy and safety of nal-IRI + 5-FU/LV in gemcitabine-refractory advanced PDAC are scarce^20–22^. Herein, we report the experience from National Cheng Kung University Hospital (NCKUH), a high-volume medical center for pancreatic cancer management in southern Taiwan^23^.

## Methods

### Patients

This retrospective study included 44 consecutive gemcitabine-refractory, advanced PDAC patients who had nal-IRI + 5-FU/LV treatment at NCKUH between December 2016 and December 2018. The median follow-up time, from the date of starting treatment to the date of cutoff at July 1st, 2019 was 19.7 months. Patient demographics, clinicopathological characteristics, dates of confirmed PDCA diagnosis and detecting metastatic disease, all previous treatments before nal-IRI + 5-FU/LV, date of starting nal-IRI + 5-FU/LV, dates of disease progression after nal-IRI + 5-FU/LV and death or last follow-up as well as all treatment-related adverse events were recorded through electronic medical records review. Generally, tumor assessment was done by computed tomography (CT) before treatment and every 12 weeks after treatment mainly based on national health insurance regulation and the in-charge physician’s discretion. Tumor response was assessed by RECIST version 1.1 and patients who presented obvious clinical disease progression were classified as having progressive disease (PD) if no follow-up images were available.

### Statistical analysis

Descriptive statistics were applied to demographics and presented as median or percentage, as appropriate. Progression-free survival (PFS) was calculated from the date of starting nal-IRI + 5-FU/LV treatment to the date of disease progression or death/loss follow-up, whichever occurred first. The time of PD was defined as the date of radiographic progression as judged by a radiologist or clinical progression warranting a change in or termination of treatment, as judged by the treating physician. Survival was determined by the Kaplan–Meier method, and differences in survival between groups were compared using the log‐rank test. All variables with *p* < 0.05 were considered to be statistically significant. All statistical analyses were performed using R version 3.5.1. This retrospective analysis was approved by the National Cheng Kung University Hospital (NCKUH) Institutional Review Board (No. A-ER-108-113) with waiver of informed consent and followed the Helsinki Declaration.

## Results

### Patient characteristics

A total of 44 patients with advanced PDAC who received at least one dose of nal-IRI + 5-FU/LV at NCKUH between December 2016 and December 2018 were identified. The baseline demographics, clinicopathological characteristics and dose delivery are listed in Table 1. Their median age was 60 years old (range 26-80) and 25 patients were male (56.8%). Baseline characteristics before starting nal-IRI + 5-FU/LV were Eastern Cooperative Oncology Group performance score (ECOG PS) 0-1 in 32 (72.7%), presence of metastatic diseases in 39 (88.6%) with 56.8% exhibiting liver metastasis and failed to one prior chemotherapy in 32 (72.7%). The most common prior regimen was gemcitabine-based triplet therapy consisting of gemcitabine, oxaliplatin plus either 5-FU/LV or S-1/LV^14,15^ in 24 (54.5%); while only 4 patients (9.1%) had prior exposure to irinotecan.

**Table 1:** Baseline demographics and clinical characteristics.

	Standard starting dose, N = 15	Reduced starting dose, N = 29*	Overall N = 44
**Gender**			
Male	8 (53.3%)	17 (58.6%)	25 (56.8%)
Female	7 (46.7%)	12 (41.4%)	19 (43.2%)
Age, median (range), years	64 (26–69)	59 (34–80)	60 (26–80)
**ECOG performance score**			
0-1	13 (86.7%)	19 (65.5%)	32 (72.7%)
≥2	2 (13.3%)	10 (34.5%)	12 (27.3%)
**Initial albumin, median (range), g/dl**	4.1 (3.2–4.4)	3.4 (1.7–4.6)	3.7 (1.7-4.6)
**Pancreatic tumor location**			
Head	6 (40.0%)	17 (58.6%)	23 (52.3%)
Body	1 (6.7%)	7 (24.1%)	8 (18.2%)
Tail	8 (53.3%)	5 (17.2%)	13 (29.5%)
**Stage at start of nal-IRI**			
III	1 (6.7%)	4 (13.8%)	5 (11.4%)
IV	14 (93.3%)	25 (86.2%)	39 (88.6%)
**Number of metastatic sites**			
0	1 (6.7%)	4 (13.8%)	5 (11.4%)
1	10 (66.7%)	19 (65.5%)	29 (65.9%)
≥2	4 (26.7%)	6 (20.6%)	10 (22.7%)
**Sites of metastatic lesions**			
Liver	10 (66.7%)	15 (51.7%)	25 (56.8%)
Peritoneum	6 (40.0%)	10 (34.5%)	16 (36.4%)
Lung	2 (13.3%)	2 (6.9%)	4 (9%)
Others	2 (13.3%)	5 (17.2%)	7 (15.9%)
**CA19-9 at start of nal-IRI treatment**			
≥40 IU/ml	11	16	27 (61.4%)
<40 IU/ml	0	3	3 (6.8%)
Not measured	5	9	14 (31.8%)
Median (range), IU/ml	894 (42.5-10000)	1070 (22.6-14210)	1016.7 (22.6-14210)
**Previous lines of therapy**			
1	13 (86.7%)	19 (65.5%)	32 (72.7%)
2	2 (13.3%)	8 (27.6%)	10 (22.7%)
3	0	2 (6.9%)	2 (4.5%)
**Distribution of first-line regimen**			
Gemcitabine, oxaliplatin plus 5-FU or S-1	12 (80.0%)	12 (41.4%)	24 (54.5%)
Gemcitabine plus S-1	1 (6.7%)	7 (24.1%)	8 (18.2%)
Gemcitabine plus nab-paclitaxel	1 (6.7%)	4 (13.8%)	5 (11.4%)
FOLFIRINOX	0	1 (3.4%)	1 (2.3%)
Gemcitabine or S-1 monotherapy	0	2 (6.9%)	2 (4.5%)
Others	1 (6.7%)	3 (10.3%)	4 (9.1%)
Prior use of irinotecan	0	4	4 (9.1%)

*Reduction of starting dose by 20% at the physician’s discretion for better tolerability.

### Drug delivery

**Figure 1:**
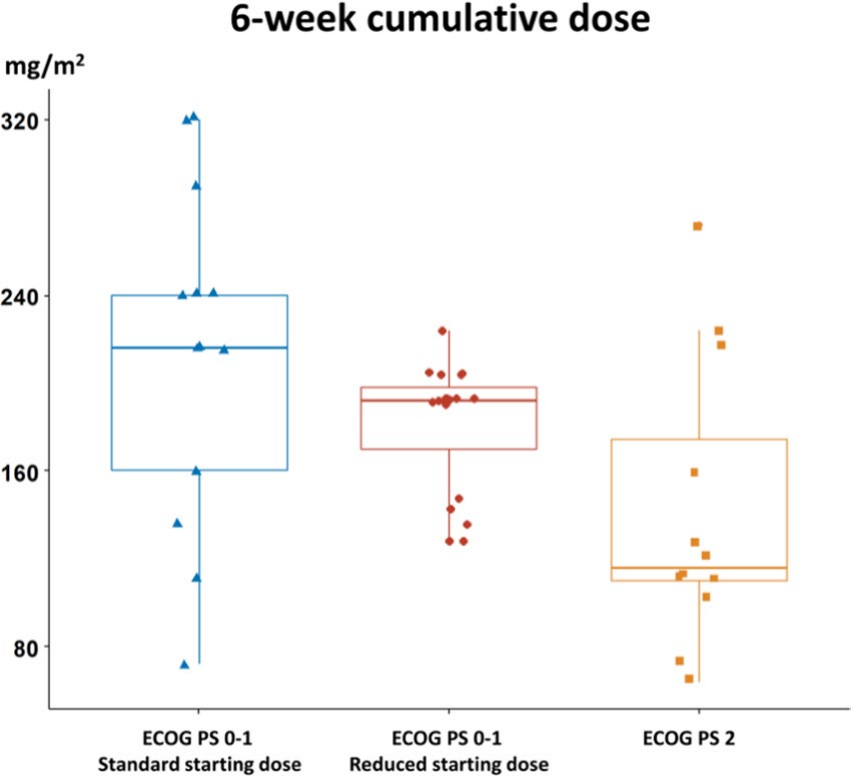
Median (line inside box), 25th and 75th percentiles (lower and upper box boundaries) of 6-week cumulative dose in ECOG PS 0-1 patients of the standard starting dose group (blue), reduced starting dose group (red) and ECOG PS 2 patients (orange).

Besides the inclusion of 12 (27.3%) patients of ECOG PS ≥ 2, the other unique feature of this study was 29 (65.9%) patients had lower initial dose of nal-IRI. The starting dose of nal-IRI was reduced by 20% to 60 mg/m^2^ (equivalent to 52 mg/m^2^ irinotecan free-base) in 19 of 32 patients with ECOG PS 0-1 and 10 of 12 patients with ECOG PS ≥ 2 aiming to improve the treatment compliance at the discretion of in-charged physicians. The median baseline albumin level of patients with the standard 80 mg/m^2^ (equivalent to 70 mg/m^2^ irinotecan free-base) and reduced 60 mg/m^2^ starting dose groups were 4.1 and 3.4 g/dl, respectively (Table 1). A summary of nal-IRI delivery is listed in Table 2. The median treatment duration and administration cycle in the standard and reduced starting dose groups was 14.6 weeks and 6 cycles, and 9.6 weeks and 4 cycles, respectively. The average interval between treatment cycles was 2.9 and 3.0 weeks in corresponding groups. Six (20.6%) patients with 60 mg/m^2^ starting dose had dose escalation to 70-80 mg/m^2^ during their treatment course. The median 6-week cumulative dose in ECOG PS 0-1 patients of standard and reduced starting dose and ECOG PS 2 were 216, 192 and 116 mg/m^2^, respectively (Fig. 1).

**Table 2:** Dose delivery.

	Standard starting dose, N = 15	Reduced starting dose, N = 29*	Overall N = 44
**Duration of treatment, weeks**			
Mean ± standard deviation	17.4 ± 15.6	18.3 ± 20.4	18.0 ± 18.7
Median (range)	14.6 (0-58.0)	9.6 (0-80.6)	9.9 (0-80.6)
**Cycle(s) received**			
Mean ± standard deviation	6.5 ± 4.8	6.8 ± 6.0	6.7 ± 5.6
Median (range)	6.0 (1-20)	4.0 (1-27)	5.0 (1-27)
**Average interval between each cycle, weeks**			
Mean ± standard deviation	2.6 ± 1.3	2.9 ± 1.0	2.83 ± 1.1
Median (range)	2.9 (0-5.3)	3.0 (0-5.3)	2.9 (0-5.3)
**Dose intensity (%)**			
Mean ± standard deviation	68.6 ± 17.9	57.7 ± 14.5	61.4 ± 16.4
Median (range)	70 (37-100)	57 (32-85)	61 (32-100)
**6-week cumulative dose, mg/m**^**2**^			
Mean ± standard deviation	204 ± 79.4	168 ± 46.7	180 ± 61.5
Median (range)	216 (72–320)	192 (64–272)	192 (64–320)
**Dose reduction in subsequent cycle(s)**	5 (33.3%)	4 (13.8%)	9 (20.5%)
**Dose escalation in subsequent cycle(s)**	0	6 (20.7%)	6 (13.6%)

*Reduction of starting dose by 20% at the physician’s discretion for better tolerability.

### Treatment response

Therapeutic efficacy is summarized in Table 3. The median PFS was 2.5 months (95% confidence interval [95% CI]: 2.2-5.8 months) and median OS was 6.6 months (95% CI: 3.8-9.5 months) for the entire cohort. The overall response rate of entire cohort was 9.1%. Two of four responders, locally advanced and metastatic PDAC in one each, who failed to SLOG had successful conversion surgery after second-line nal-IRI + 5-FU/LV. Both patients achieved more than one year of relapse-free survival after surgery.

**Table 3:** Treatment efficacy.

	Stage IV only N = 39	Overall N = 44
**PFS (months), median (95% CI)**	2.5 (2.0-4.8)	2.5 (2.2-5.8)
**OS (months), median (95% CI)**	6.0 (3.2-8.4)	6.6 (3.8-9.5)
**Best overall response**		
Partial response	2 (5.1%)	4 (9.1%)
Stable disease	13 (33.3%)	15 (34.1%)
Progressive disease*	24 (61.5%)	25 (56.8%)
**OS from confirmed metastatic disease, median (95% CI)**	13.2 (10.4-25.6)	—

*Includes clinical progressive disease lacking CT scan evaluation.

**Figure 2:**
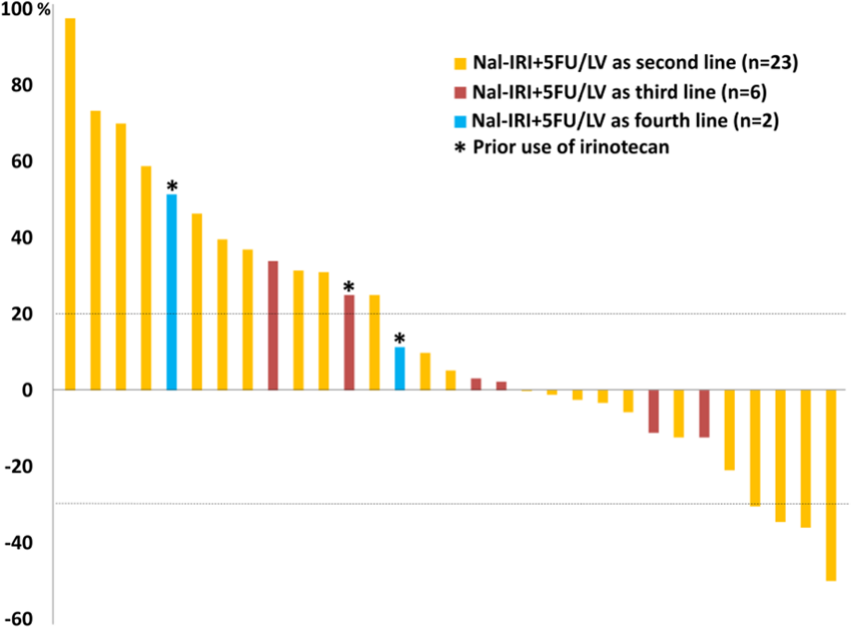
Waterfall plots demonstrate the percentage change of overall tumor size after nal-IRI treatment; different colors indicate different line settings.

**Figure 3:**
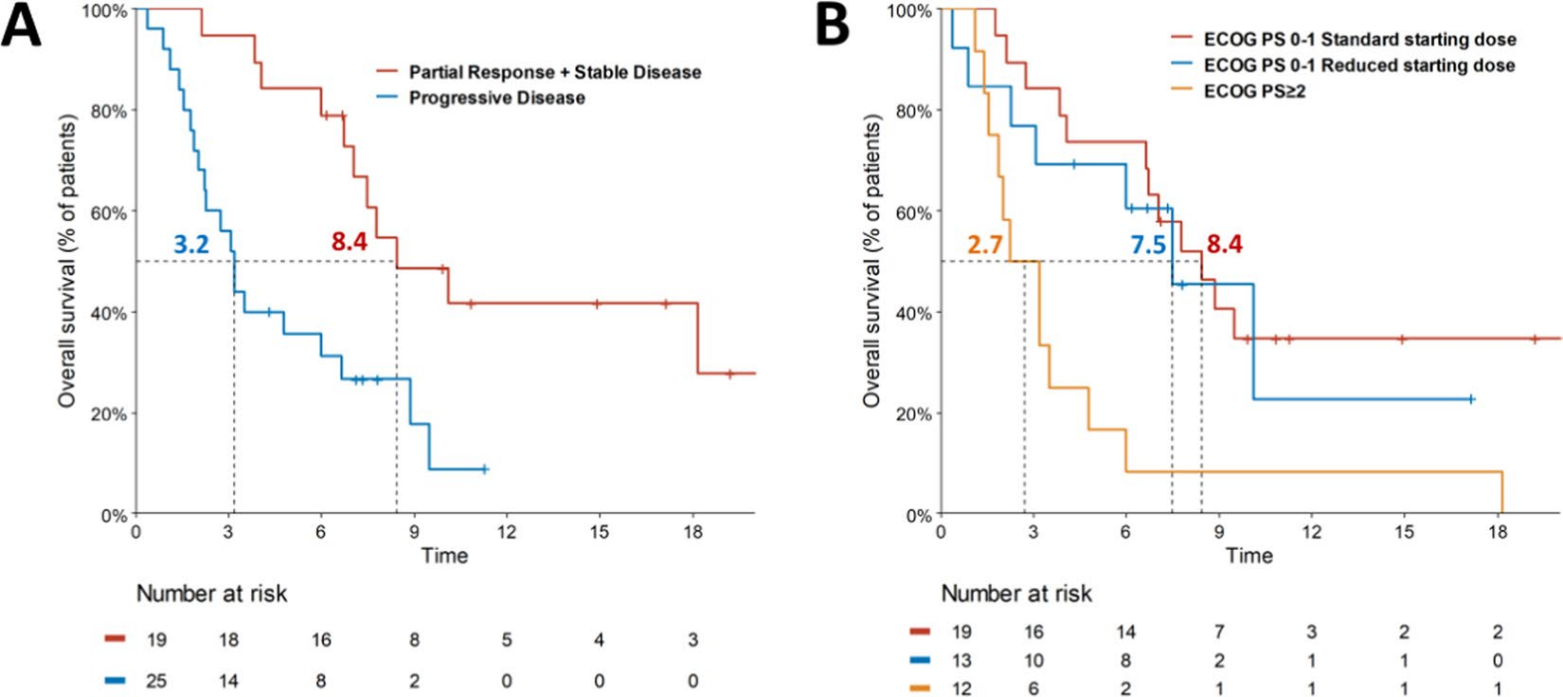
Kaplan-Meier survival analyses of (**A**) median overall survival by best overall response (**B**) median overall survival of ECOG PS 0-1 with/without initial dose reduction and ECOG PS ≥ 2.

A total of 31 patients had evaluable target lesions; the percentage of change in tumor size is depicted in Fig. 2. Of the entire cohort, the median OS was 8.4 months in patients with partial response (PR) or stable disease (SD), and 3.2 months in the PD group (Fig. 3A). The median OS was 7.8 months for the 32 ECOG PS 0-1 patients and 2.7 moths for the 12 ECOG PS ≥ 2 patients. The median OS of the ECOG PS 0-1 patients with and without initial dose reduction were 8.4 and 7.5 months, respectively (*p* = 0.95), as shown in Fig. 3B.

**Figure 4:**
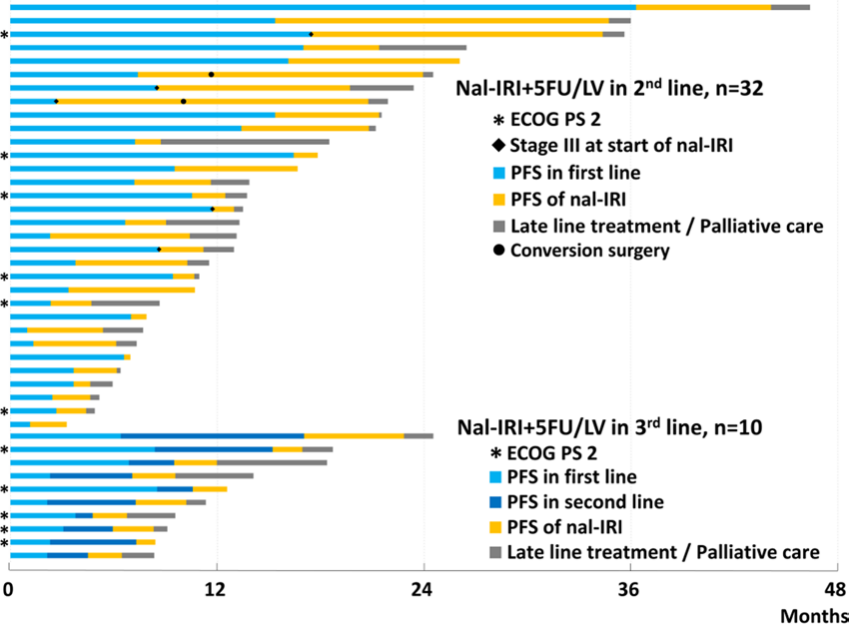
Swimmer plot demonstrating duration of therapy in months; different colors represent different lines of therapy in each patient.

The duration of treatments before and after nal-IRI + 5-FU/LV was available in 42 patients. Twenty-four out of 42 patients (57.1%) with advanced PDAC survived for more than one year, calculated from the start of first-line treatment (Fig. 4). The median OS after the first radiological evidence of metastatic diseases was 13.2 months (95% CI: 10.4-25.6 months) in the 39 patients with metastatic PDAC.

### Safety profiles

Safety profiles that were extracted from electronic medical records included patient symptoms, physical examination, complete blood count and biochemistry lab data (Table 4). A total of 19 patients (43.2%) experienced neutropenia, including 12 patients (27.3%) having grade 3-4 neutropenia. Only one case of febrile neutropenia (2.3%) was reported. All grades and grade 3-4 anemia were observed in 37 patients (84.1%) and 15 patients (34.1%), respectively. Thrombocytopenia was less common, with an incidence of 22.7% for all grades and 13.6% for grade 3-4. Up to 36.4% of patients experienced alanine aminotransferase (ALT) or bilirubin increases. Four patients (9.1%) had grade 3-4 bilirubin increase; however, all instances occurred concurrently with biliary tract infection. Diarrhea of any grade occurred in 36.4% of patients, with no grade 3-4 diarrhea recorded.

**Table 4:** Toxicity.

	Standard starting dose, N = 15	Reduced starting dose, N = 29	Overall N = 44
**Neutropenia**			
Any grade	6 (40.0%)	13 (44.8%)	19 (43.2%)
Grade 3-4	5 (33.3%)	7 (24.1%)	12 (27.3%)
Febrile neutropenia	1 (6.7%)	0	1 (2.3%)
**Anemia**			
Any grade	14 (93.3%)	23 (79.3%)	37 (84.1%)
Grade 3-4	5 (33.3%)	10 (34.5%)	15 (34.1%)
**Thrombocytopenia**			
Any grade	5 (33.3%)	5 (17.2%)	10 (22.7%)
Grade 3-4	4 (26.7%)	2 (6.9%)	6 (13.6%)
**Alanine aminotransferase increased**			
Any grade	4 (26.7%)	12 (41.4%)	16 (36.4%)
Grade 3-4	1 (6.7%)*	1 (3.4%)*	2 (4.5%)*
**Blood bilirubin increased**			
Any grade	4 (26.7%)	10 (34.5%)	14 (31.8%)
Grade 3-4	1 (6.7%)	3 (10.3%)*	4 (9.1%)*
**Creatinine increased**			
Any grade	2 (13.2%)	3 (10.3%)	5 (11.4%)
Grade 3-4	0	0	0
**Diarrhea**			
Any grade	4 (26.7%)	12 (41.4%)	16 (36.4%)
Grade 3-4	0	0	0

*These grade 3-4 toxicities occurred concurrently with biliary tract infection.

## Discussion

Current study shows that nal-IRI + 5-FU/LV regimen could achieve a 9.1% of response rate and 6.6 months of overall survival for gemcitabine-refractory advanced PDAC on intent-to-treat analysis in a real-world Taiwanese cohort, including 27.3% ECOG PS > 2 patients and 65.9% having reduced initial dose of nal-IRI to 60 mg/m^2^. The treatment outcomes largely concur with those of pivotal NAPOLI-1 trial in which most patients were ECOG PS 0-1 and had standard starting dose of 80 mg/m^2^ nal-IRI^16^. After excluding those with ECOG PS > 2, the median OS of our ECOG PS 0-1 cohort was 7.8 months, which was compatible with that of 8.9 months in Asian subgroup of NAPOLI-1^24^. To the best of our knowledge, the clinical outcomes of ECOG PS > 2 patients receiving nal-IRI + 5-FU/LV remains yet illustrated, in neither previous reports from the Memorial Sloan Kettering Cancer Center (MSKCC) and NAPOLI-1 studies that including 21% and 9% of ECOG PS 2 patients, respectively^16,20^. In this single-institution analysis, the median OS of 2.7 months for ECOG PS ≥ 2 group suggests that although nal-IRI + 5-FU/LV could be well tolerated, but its routine use in patients of gemcitabine-refractory advanced PDAC with ECOG PS ≥ 2 should not be encouraged.

In the pre-specified extended analysis of NAPOLI-1 study, only 41.2% of Asian patients with nal-IRI + 5-FU/LV could have 80% or more of scheduled dose intensity during the first 6 weeks of treatment as 65.3% non-Asian patients did^25^. In addition, a *post hoc* analysis of NAPOLI-1 showed Asian patients with nal-IRI + 5-FU/LV had a 54.5% of grade 3-4 neutropenia as compared to the 17.8% in Caucasian patients^24,26^. Considering the higher incidence of hematological toxicities and thus more dose modification in the nal-IRI + 5-FU/LV treated Asian population of NAPOLI-1 study^24–27^, our physicians chose to give lower starting doses of nal-IRI at 60 mg/m^2^ in some patients with poor physical performance and/or poor nutrition status. This is reflected by the fact that patients in the reduced dose group were more of ECOG PS > 2, 34.5% *versus* 13.3%, and had lower baseline median albumin level, 3.4 g/dl *versus* 4.1 g/dl, as compared to those in the standard dose group. The strategy seems to work because among those with 60 mg/m^2^ nal-IRI starting dose, only 13.8% patients required further dose reduction, while 20.6% patients could have dose escalation to 70–80 mg/m^2^ in their subsequent treatment cycle(s). Although mean dose intensity of 57.7% in the 60 mg/m^2^ starting dose group was lower than that of 68.8% in standard starting dose group, however, the survival of ECOG PS 0-1 patients with and without starting dose reduction was compatible (Fig. 3B). The impact of early dose modification has also been evaluated in a *post hoc* analysis of NAPOLI-1, in which all non-UGT1A1*6 homozygous patients received standard starting dose of nal-IRI. Among the 93 patients who had >6 weeks of scheduled nal-IRI + 5-FU/LV treatment, the median OS of those with and without early dose modification (reduction and/or delay within the first 6 weeks of treatment) was 8.4 and 8.3 months, respectively^28^. These data highlights that early dose modification of nal-IRI may not significantly affect the clinical outcomes of patients having nal-IRI + 5-FU/LV for gemcitabine-refractory advanced PDAC, regardless of the modification was made at or after the start of nal-IRI + 5-FU/LV treatment.

Since nal-IRI is a new formulation of irinotecan, it is conceived that patients with history of prior irinotecan exposure might be less likely to benefit from nal-IRI-based treatment. In both the NAPOLI-1 and a MSKCC studies, irinotecan-naïve patients were benefited more from nal-IRI + 5-FU/LV treatment as compared to those with prior irinotecan exposure with HR of 0.62 (95% CI: 0.44-0.86) and 0.38; 95% CI: 0.2-0.72), respectively^16,20^. These findings raise the concern regarding the clinical relevant of nal-IRI + 5-FU/LV in daily practice when FOLFIRINOX appears to be one of recommended first-line regimens for chemo-naïve, ECOG PS 0-1 advanced PDAC patients in all recent NCCN, ASCO and ESMO pancreatic cancer management guidelines^7–9,11^. However, due to its association with high hematological toxicity, first-line FOLFIRINOX could only be applied in 20–40% of advanced PDAC patients with good performance, as demonstrated in several recent real world population analyses^29–31^. On the other hand, gemcitabine-based regimens remain the treatment of choice for the rest of 50–60% chemo-naïve advanced PDAC patients. Among them, the use of gemcitabine plus nab-paclitaxel reached 40% after 2014 in an US population study, 47% in a Pan-European Questionnaire Study and 56.8% in a Japanese study that evaluated the dynamical changes of treatment patterns in real-world practice^29–31^. Nal-IRI + 5-FU/LV remains the only FDA and EMEA approved regimen for advanced PDAC patients who have failed to gemcitabine-based therapy.

In the past, surgery did not improve the survival of those highly selected patients with solitary liver metastasis and thus rarely considered as an treatment option for patients with metastatic PDAC^32^. With the advance of front-line multi-agent combination regimens, there is an increasing interest in conversion surgery for patients with locally advanced PDAC and very occasionally for patients with metastatic PDAC^33,34^. In current study, two of four patients with partial response after nal-IRI + 5-FU/LV treatment was able to undergo successful conversion surgery followed by maintenance nal-IRI + 5-FU/LV. Both achieved more than one year of relapse-free survival after surgery. Such experience suggests the feasibility and potential benefit of conversion surgery in advanced/metastatic PDAC patients even after second-line treatment.

Of patients in the nal-IRI + 5-FU/LV arm of the pivotal NAPOLI-1 study, Asian cohort experienced higher incidence of grade 3-4 neutropenia but lower incidence of grade 3-4 diarrhea as compared to the overall study population^16,24^. Primary granulocyte colony-stimulating factor prophylaxis was not routinely administrated in our institute. The 27.4% of grade 3-4 neutropenia in our patients was lower than that of 54.5% in the NAPOLI-1 Asian cohort, which can be partially attributed to the use of reduced starting dose of nal-IRI in 65.9% of our patients as well as lower mean dose intensity of nal-IRI in our patients, 57.7–68.6% *versus* 74.9% in the NAPOLI-1 Asian cohort^24^. None of our patients experienced grade 3-4 diarrhea further supports the observation that nal-IRI-based therapy-related grade 3-4 diarrhea less frequently occurs in Asian population as compared to non-Asian and its incidence is nal-IRI dose dependent regardless of ethnicity in earlier studies^26,27^. For instance, despite same dose intensity of nal-IRI in nal-IRI monotherapy (120 mg/m^2^, every 3 weeks) and nal-IRI + 5-FU/LV (80 mg/m^2^, every 2 weeks), the incidence of grade 3-4 diarrhea was higher in nal-IRI monotherapy, 21.1% *versus* 12.8% in overall population and 16.0% versus 3.0% in Asian population of NAPOLI-1 study^24^. A population pharmacokinetic analysis of earlier nal-IRI studies found the ethnic difference of grade 3-4 neutropenia and diarrhea can be associated with the Cmax level of uncapsulated SN38 and total irinotecan, respectively^26^. Unfortunately, the genetic background under such pharmacokinetic differences remain largely unknown and deserve further investigation.

There are some limitations in our retrospective study that are also common in other real-world data analysis. First, the inclusion of fragile patients of ECOG PS > 2; secondly, more labile treatment and dose modification schedule in daily practice; and thirdly, data extraction from electronic medical records which may not comprehensive and accurate as those from clinical trials. However, our study not only reflects the experience of real-world practice in a high-volume medical center in Taiwan, but also provides evidence for the feasibility and efficacy of nal-IRI + 5-FU/LV for ECOG PS > 2 patients.

## Conclusion

Our study confirmed the therapeutic efficacy of nal-IRI + 5-FU/LV treatment with acceptable toxicity profiles in a Taiwanese cohort. However, the benefits for patients with ECOG PS ≥ 2 remained unremarkable. Our experience supports the claim that nal-IRI + 5-FU/LV could serve as standard care after the failure of first-line gemcitabine-based treatment in patients with advanced PDAC in real-world clinical settings.
