# Author Correction: The Impact of Liposomal Irinotecan on the Treatment of Advanced Pancreatic Adenocarcinoma: Real-World Experience in a Taiwanese Cohort

**DOI:** 10.1038/s41598-020-77250-4

**Published:** 2020-11-13

**Authors:** Yung-Yeh Su, Nai-Jung Chiang, Hui-Jen Tsai, Chia-Jui Yen, Yan‐Shen Shan, Li‐Tzong Chen

**Affiliations:** 1 National Institute of Cancer Research, National Health Research Institutes, Tainan, Taiwan; 2 Division of Hematology and Oncology, Department of Internal Medicine, National Cheng Kung University Hospital, College of Medicine, National Cheng Kung University, Tainan, Taiwan; 3 Institute of Clinical Medicine, College of Medicine, National Cheng Kung University, Tainan, Taiwan; 4 College of Medicine, National Cheng Kung University, Tainan, Taiwan; 5 Department of Internal Medicine, Kaohsiung Medical University Hospital and Kaohsiung Medical University, Kaohsiung, Taiwan

Correction to: *Scientific Reports* https://www.doi.org/10.1038/s41598-020-64421-6, published online 04 May 2020

This Article contains a typographical error in the Discussion section where all instances of “ECOG PS $>$ 2” should read “ECOG PS $\ge$ 2”.

